# TRAF6 Lactylation in Glycolytic Macrophages Drives NF‐κB Signaling and M1 Polarization During Orthodontic Tooth Movement

**DOI:** 10.1002/advs.76518

**Published:** 2026-07-14

**Authors:** Xinyi He, Guodong Zhao, Yun Hu, Wenya Bai, Hao Tan, Shuai Yuan, Sihan Yang, Ye Zhu, Xiaohui Xu, Leilei Zheng

**Affiliations:** ^1^ College of Stomatology Chongqing Medical University Chongqing China; ^2^ Chongqing Key Laboratory of Oral Diseases Chongqing China; ^3^ Chongqing Municipal Key Laboratory of Oral Biomedical Engineering of Higher Education Chongqing China; ^4^ Chongqing Key Laboratory of Big Data for Bio Intelligence Chongqing University of Posts and Telecommunications Chongqing China; ^5^ Institute of Stomatology School and Hospital of Stomatology Wenzhou Medical University Wenzhou Zhejiang China

**Keywords:** bone remodeling, inflammation, lactate dehydrogenases, metabolic reprogramming, molecular dynamics simulations, posttranslational modifications

## Abstract

Protein lactylation is an emerging lactate‐derived modification, coupling metabolic reprogramming to inflammatory regulation while modulating cellular responses to the microenvironment. However, the role of macrophage lactylation in orthodontic tooth movement (OTM) remains unclear. Transcriptomic and metabolomic profiling of compressed macrophages identified glycolytic reprogramming as the core regulatory axis, with elevated lactate levels validated in macrophages, mice, and human saliva. Exogenous lactate promoted M1 polarization and NF‐κB signaling activation in compressed macrophages, identifying lactate dehydrogenase A (LDHA) as a critical regulatory node. Myeloid‐specific *Ldha* deficiency inhibited OTM and attenuated sterile inflammation, confirming the critical link between lactate metabolism and OTM progression. Mechanistically, we utilized lactylation proteomics and identified that lactate induces specific lactylation of TRAF6 at lysine residues 171, 180, and 388 (K171, K180, K388). Molecular dynamics simulations and site‐directed mutagenesis revealed that K171/K180 lactylation enhances TRAF6 K63‐linked ubiquitination, thereby driving NF‐κB signaling activation. Consistently, mutation of K171 and K180 diminished TRAF6 K63‐linked ubiquitination and suppressed NF‐κB activation. Collectively, our findings demonstrate that sustained compressive force reprograms macrophage metabolism toward glycolysis and drives lactylation of TRAF6 at K171/K180, which serves as a core regulatory node that amplifies NF‐κB signaling, thereby facilitating OTM‐associated sterile inflammation and alveolar bone remodeling.

## Graphical abstract

Schematic diagram showing compressive stress triggers glycolytic reprogramming and lactate accumulation in macrophages. Lactate mediates TRAF6 lactylation at K171/K180 dual sites, which enhances its K63‐linked ubiquitination to activate the NF‑κB pathway and promote M1 polarization. This cascade drives orthodontic tooth movement (OTM) and alveolar bone remodeling, highlighting a metabolic – epigenetic axis in mechanobiology.

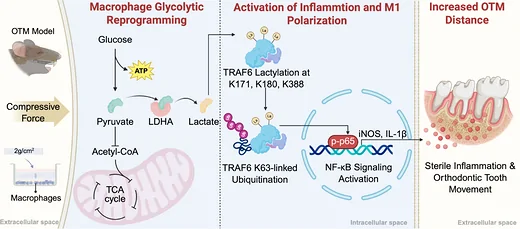

## 1 Introduction

Orthodontic tooth movement (OTM) is fundamentally driven by the application of mechanical force, which initiates a tightly orchestrated cascade of periodontal tissue remodeling events, including alveolar bone resorption and formation [1]. This adaptive process critically depends on a localized sterile inflammatory response governed by the innate immune system, a phenomenon increasingly conceptualized as mechano‐immunity [2, 3]. Among innate immune cells, macrophages function as highly sensitive mechanosensors and play indispensable roles in initiating, amplifying, and resolving inflammatory responses during OTM [4]. Notably, He et al. demonstrated that M1 macrophage infiltration peaks at day 3 following orthodontic force application and is essential for osteoclast activation and OTM initiation, whereas monocyte/macrophage depletion markedly impairs tooth movement [5]. Under mechanical stimulation, macrophages preferentially acquire a pro‐inflammatory (M1‐like) phenotype and secrete a broad spectrum of inflammatory cytokines, thereby promoting osteoclastogenesis and modulating the activity of other periodontal ligament (PDL) cells [6, 7]. However, the molecular mechanisms by which macrophages perceive mechanical cues and translate them into inflammatory signaling programs during OTM remain poorly defined.

Accumulating evidence indicates that mechanical force induces profound metabolic reprogramming across multiple periodontal cell types during OTM [8]. For example, Liu et al. reported that mechanical stimulation enhances both glycolysis and oxidative phosphorylation (OXPHOS) in periodontal osteoprogenitors through a PGC‐1α/LDHA‐dependent pathway [9]. In parallel, Zhang et al. demonstrated that orthodontic force drives periodontal ligament stem cells (PDLSCs) toward anaerobic metabolism [10]. These findings collectively suggest that mechanical stress promotes a glycolytic shift—characterized by preferential reliance on glycolysis—commonly referred to as glycolytic reprogramming [11]. This metabolic adaptation has emerged as a key driver of pathological and reparative processes in diverse conditions, including pulmonary fibrosis, heart failure, atherosclerosis, and oral diseases such as periodontal remodeling and OTM [12, 13]. Importantly, glycolytic reprogramming has been shown to actively promote inflammatory activation and M1 polarization of macrophages [14], whereas stress relaxation–associated suppression of glycolysis favors a reparative macrophage phenotype [15]. These observations raise the possibility that force‐induced glycolytic reprogramming may represent a critical metabolic mechanism driving macrophage inflammatory activation during OTM, although the underlying regulatory pathways remain largely unexplored.

One principal mechanism by which glycolysis influences immune cell function is through the generation of lactate and subsequent lysine lactylation. Protein lactylation is a lactate‐derived post‐translational modification that functions as a metabolic sensor, directly linking microenvironmental metabolic states to epigenetic and functional reprogramming in immune cells [16]. Previous studies have shown that enhanced glycolytic flux promotes histone H3K18 lactylation (H3K18la) in macrophages, thereby amplifying inflammatory gene expression programs in hypoxia‐ and infection‐associated diseases [17, 18]. Nevertheless, the global landscape of macrophage protein lactylation during OTM and the key lactylated proteins that govern macrophage inflammatory phenotypes under mechanical stress remain unknown.

In the present study, we investigated how compressive force reprograms macrophage immunometabolism during OTM. We demonstrate that mechanical loading enhances lactate production via LDHA, leading to a broad remodeling of the macrophage protein lactylation profile. *Ldha* knockout in macrophages leads to reduced NF‐κB signaling and M1 polarization. Mechanistically, we identify TRAF6 as a previously unrecognized lactylation substrate and show that TRAF6 lactylation promotes its K63‐linked ubiquitination, thereby activating NF‐κB signaling and driving M1 macrophage polarization in the mechanically stressed periodontium. Our findings reveal a metabolic–post‐translational modification axis linking mechanical force to macrophage inflammatory activation and suggest that targeting macrophage lactate metabolism and TRAF6 lactylation may represent a promising strategy to modulate OTM.

## 2 Results

### 2.1 Compressive Loading Induces Glycolytic Reprogramming and Promotes Lactate Accumulation in Macrophages

To characterize the metabolic alterations of macrophages under mechanical loading, we established an in vitro model in which THP‐1 macrophages were subjected to mechanical compression (2.0 g/cm^2^) for 24 h (Figure 1a). Transcriptomic profiling by RNA‐seq (Figure S1a) revealed an extensive divergence in gene expression between control and compressed THP‐1 macrophages (Figure S1b). KEGG pathway analysis of differentially expressed genes demonstrated an enhanced enrichment of the glycolytic activity pathway, accompanied by NF‐κB signaling pathway enrichment (Figure 1b). Consistently, GSEA further verified coordinated activation of glycolysis and NF‐κB pathways under mechanical compression (Figure 1c), indicating that macrophages undergo glycolytic reprogramming in parallel with inflammatory signaling activation. At the protein level, Western blot analysis corroborated these findings, showing enhanced NF‐κB signaling activation and M1 polarization, as demonstrated by an increased p‐p65/p65 ratio and elevated expression of iNOS and CD86 (Figure S1c).

**FIGURE 1:**
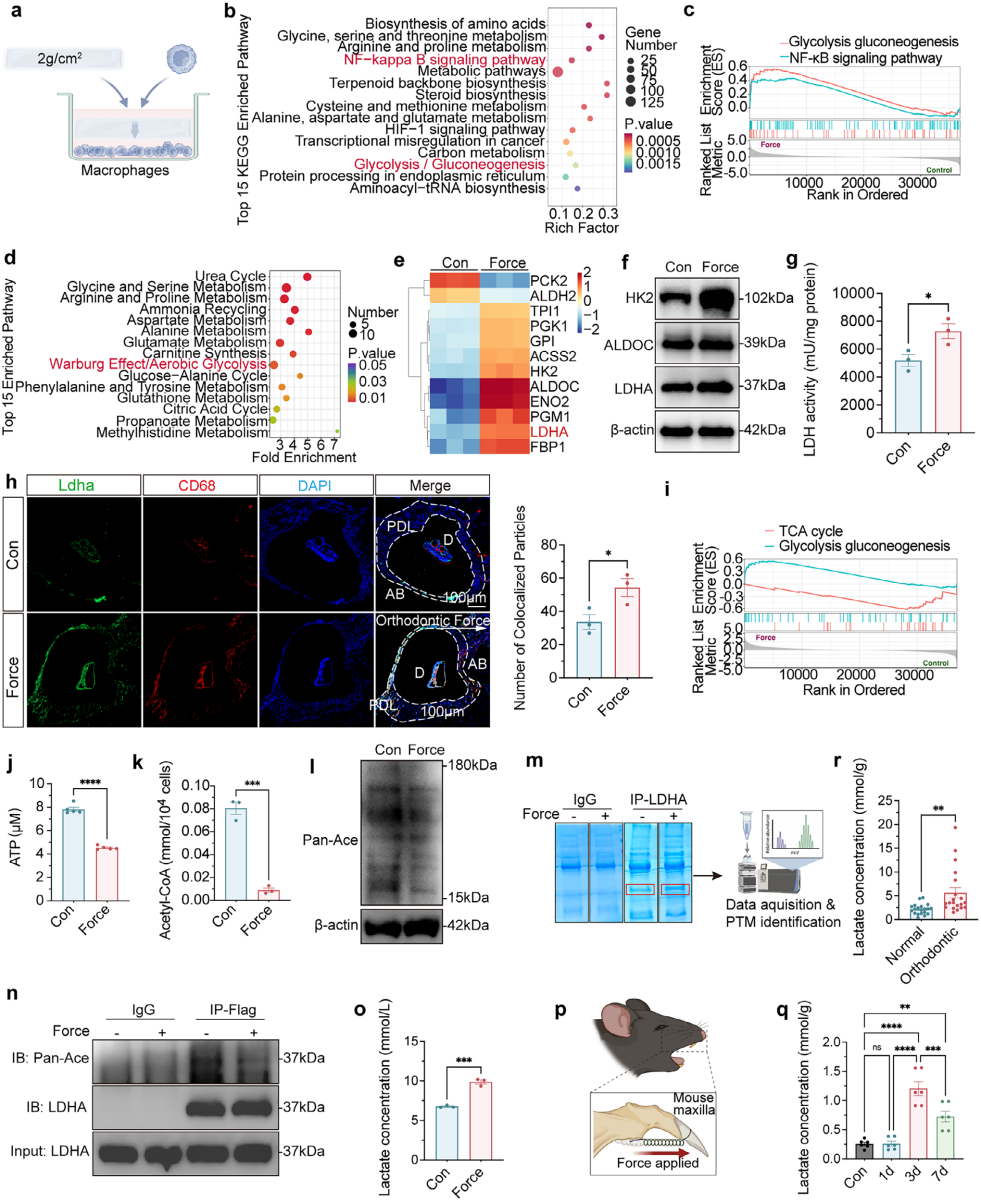
Compressive loading induces glycolytic reprogramming and lactate production in macrophages. (a) Schematic diagram of the in vitro compressive loading model. THP‐1 cells were subjected to physiological compression (2.0 g/cm^2^) for 24 h. (b) The glycolysis and NF‐κB signaling pathways were enriched in the KEGG enrichment analysis of DEGs from RNA‐seq. (c) GSEA analysis of the glycolysis and NF‐κB signaling pathways. (d) The aerobic glycolysis pathway was enriched in the KEGG enrichment of differential metabolites. (e) Heatmap of glycolysis‐associated genes. (f) Protein levels of glycolytic enzymes in THP‐1 macrophages with or without compressive loading. (g) LDH enzymatic activity of THP‐1 treated with or without compression. (h) Co‐immunofluorescence of Ldha and CD68 in mice periodontal tissues. (i) GSEA analysis of the glycolysis and TCA cycle pathways. (j, k) ATP (j) and acetyl‐CoA (k) concentrations in THP‐1 cells (*n* = 3). (l) Western blot analysis of Pan‐Ace level in macrophages with or without compressive force. (m) MS‐based quantitative analysis of lysine acetylation sites on LDHA protein under compressive force. (n) THP‐1 cells were transfected with lentivirus encoding Flag‐*LDHA* for 24 hours, followed by physiological compression or not. Cell extracts were subjected to immunoprecipitation (IP) with the IgG or an antibody against Flag. Co‐IP analysis of the *LDHA* acetylation level was determined. (o) The lactate concentrations in THP‐1 cells (*n =* 3). (p) Schematic diagram of OTM in mice. (q) The lactate concentrations in mouse periodontal tissues at the indicated time (*n* = 6). (r) The lactate concentrations in saliva from healthy controls and participants who underwent orthodontic (*n* = 10). OTM, orthodontic tooth movement. Data are presented as mean ± SD. ^**^p < 0.01, ^***^p < 0.001, ^****^p < 0.0001.

To further delineate the metabolic consequences of mechanical compression, we performed Q300 metabolomic profiling. Principal metabolite differences were observed between compressed and control macrophages (Figure S1d), and KEGG pathway analysis of differentially abundant metabolites revealed significant enrichment of the aerobic glycolysis pathway in response to compression (Figure 1d).

We next analyzed expression levels of glycolysis‐related genes. Heatmap visualization of these genes showed upregulation of multiple glycolytic enzymes, including LDHA, the key enzyme of lactate production (Figure 1e). We verified the increased glycolytic‐associated protein, including HK2 and ALDOC (Figure 1f; Figure S1e). Direct measurement of LDH enzymatic activity confirmed that mechanical compression significantly increased its catalytic activity (Figure 1g). However, LDHA protein levels were only modestly increased under compression (Figure 1f; Figure S1e). Consistent with the in vitro findings, only a mild increase in Ldha expression was observed in CD68+ macrophages within the periodontal compression region in vivo (Figure 1h). This modest elevation in LDHA protein levels was insufficient to account for the pronounced increase in glycolytic flux and LDH enzymatic activity. Because LDHA activity is negatively regulated by acetylation—and acetylation depends on acetyl‐CoA produced by the TCA cycle—we next assessed acetylation‐related metabolic changes [19, 20]. Mechanical compression suppressed the TCA cycle and protein acetylation in THP‐1 macrophages, as demonstrated by GSEA analysis from RNA‐seq, and significantly reduced ATP production (Figure 1i,j; Figure S1f). Moreover, compression caused a global reduction in acetyl‐CoA levels and protein acetylation (Figure 1k,l; Figure S1g). We next investigated the acetylation level of LDHA by MS‐based quantitative analysis (Figure 1m). As expected, LDHA acetylation was markedly decreased, consistent with enhanced LDH enzymatic activity (Figure 1n,g; Figure S1h,i).

We evaluated lactate accumulation as a functional outcome of glycolytic reprogramming. Mechanical compression significantly increased intracellular lactate levels in macrophages (Figure 1o). In vivo, a murine orthodontic tooth movement (OTM) model using 20 g nickel–titanium springs demonstrated that periodontal lactate concentrations peaked at day 3 after force application (Figure 1p,q), with the increased OTM distance, enhanced NF‐κΒ signaling, and M1 polarization (Figure S1j–l). Clinically, salivary lactate levels in orthodontic patients were approximately two‐fold higher than those in control subjects (Figure 1r). Collectively, these findings indicate that compressive loading induces metabolic reprogramming in macrophages—characterized by enhanced glycolysis, reduced acetylation, and elevated lactate production—which may serve as a key driver of force‐induced inflammatory activation.

### 2.2 Lactate Positively Regulates NF‐κB Signaling Pathway and Promotes M1 Polarization of Macrophage

To determine whether lactate contributes to inflammatory activation in macrophages under compressive force, THP‐1 cells were treated with lactate. A concentration of 10 mm was selected because compressive force increased intracellular lactate levels to approximately 10 mm (Figure 1o), consistent with levels reported in inflammatory microenvironments [16]. Moreover, 10 mm lactate did not affect cell viability, whereas higher concentrations (20–40 mm) significantly reduced cell survival (Figure S2a). NF‐κB signaling and M1 polarization were assessed by examining the p‐p65/p65 ratio as well as the expression of iNOS and CD86. As shown in Figure 2a and Figure S2b, lactate markedly increases the levels of these markers, indicating that lactate is sufficient to promote a pro‐inflammatory macrophage phenotype. In vivo, local lactate injection into the murine periodontium accelerated orthodontic tooth movement (Figure 2b,c) and enhanced the infiltration of iNOS+CD68+ macrophages in periodontal tissues (Figure 2d). This was accompanied by strengthened p‐p65 expression in CD68+ cells (Figure 2e). These findings demonstrate that lactate acts as an active inflammatory mediator, capable of triggering NF‐κB‐mediated signaling, driving macrophage M1 polarization, and promoting force‐induced bone remodeling.

**FIGURE 2:**
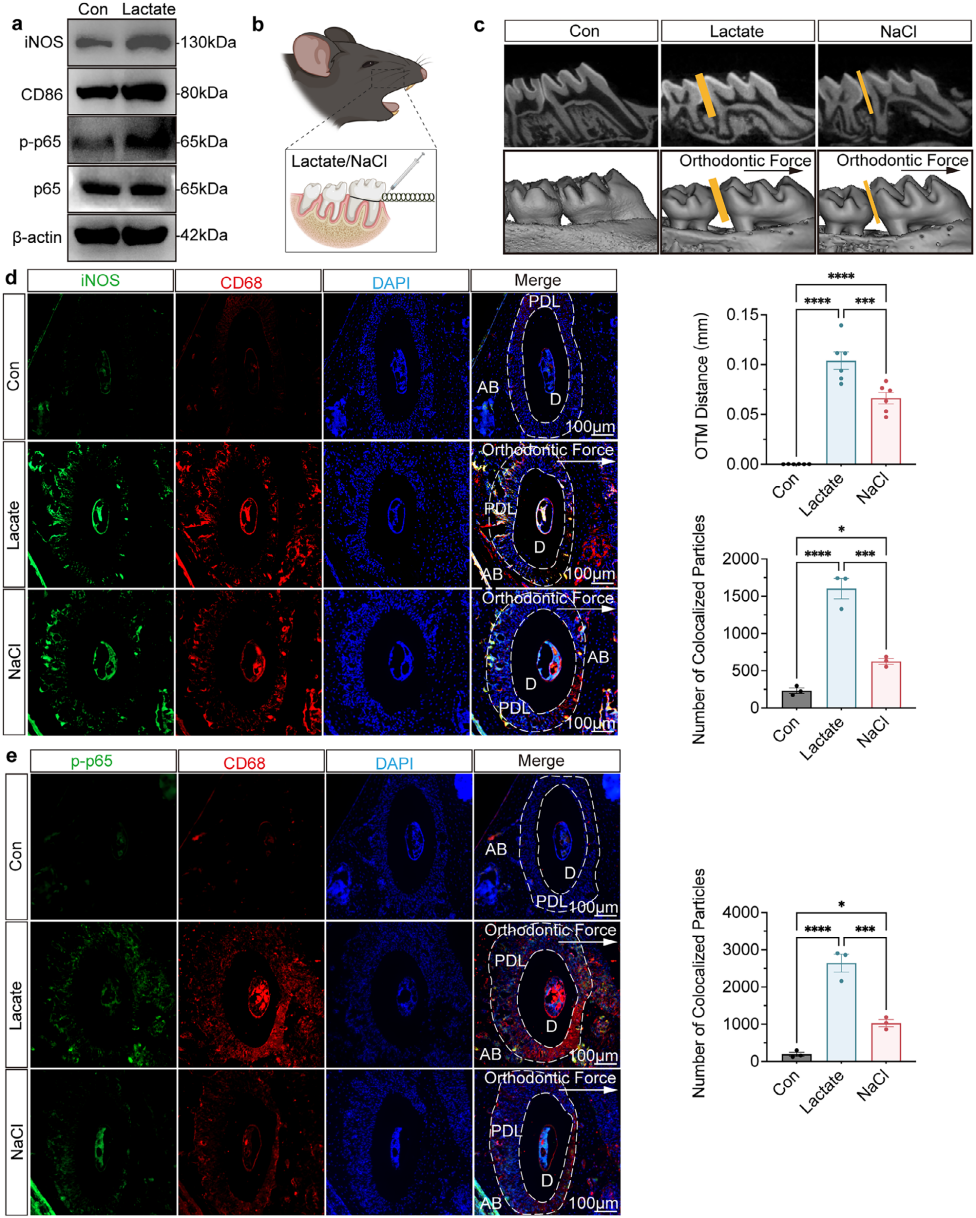
Lactate positively regulates NF‐κB signaling pathway and promotes M1 polarization of macrophages. (a) Protein levels of p65, p‐p65, iNOS, and CD86 in THP‐1 cells treated with or without lactate (10 mm). (b) Schematic diagram of the study. The mouse OTM model was subjected to periodontal injection of lactate/NaCl. (c) 3D micro‐CT reconstructed images (sagittal view) of maxillae from controls and mice with periodontal injection of lactate/NaCl. OTM distances were quantified (*n* = 6). (d) Co‐immunofluorescence of iNOS and CD68 in mouse periodontal tissues (*n* = 3). (e) Co‐immunofluorescence of p‐p65 and CD68 in mouse periodontal tissues (*n* = 3). OTM, orthodontic tooth movement. Data are presented as mean ± SD. ^*^p < 0.05, ^***^p < 0.001, ^****^p < 0.0001.

### 2.3 *Ldha* Knockout in Macrophage Attenuates NF‐κB Signaling and M1 Polarization During Orthodontic Tooth Movement

To further decipher the contribution of macrophage glycolytic flux to orthodontic tooth movement, we first silenced *LDHA* (si‐*LDHA*) in THP‐1 cells under mechanical force (Figure S3a). As expected, *LDHA* knockdown under compression resulted in a pronounced reduction in global lactylation (Figure 3a) and diminished lactate generation (Figure S3b). Functionally, *LDHA* knockdown under compression markedly decreased mRNA expression of *iNOS* and *IL‐1β* (Figure 3b), along with a reduced p‐p65/p65 ratio and decreased protein expression of iNOS and IL‐1β (Figure 3c; Figure S3c), indicating suppression of NF‐κB signaling, M1 polarization, and pro‐inflammatory cytokine production.

**FIGURE 3:**
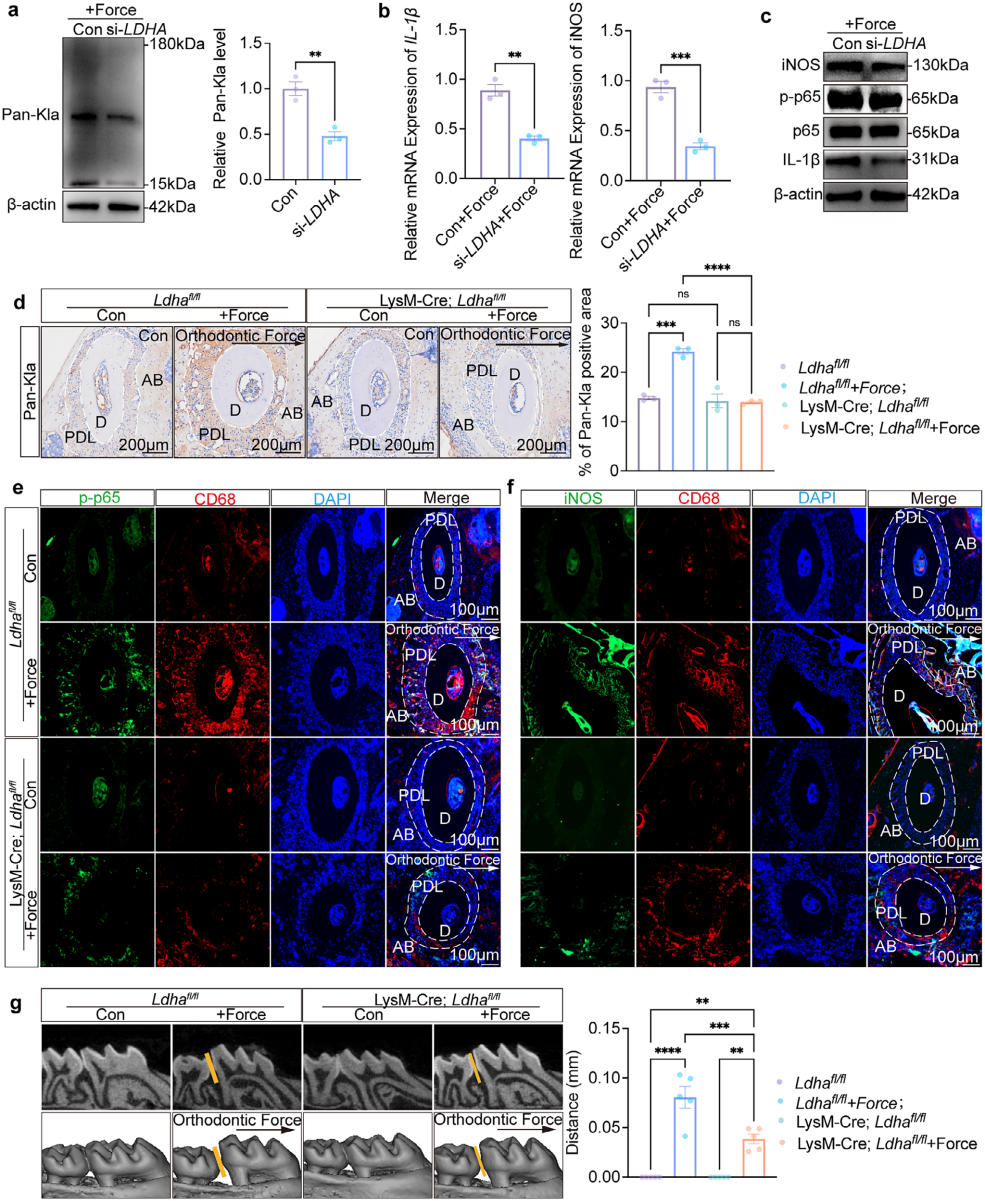
*Ldha* knockout in macrophage attenuates NF‐κB signaling and M1 polarization during OTM. (a) THP‐1 cells were transfected with si‐Con/*LDHA*, followed by a Western blot for pan‐lactylation (Pan‐Kla) levels (*n* = 3). (b) The mRNA level of *IL‐1β* and *iNOS* in THP‐1 cells transfected with si‐Con/*LDHA* under compression (*n* = 3). (c) Western blot analysis of M1 marker (iNOS), NF‐κΒ signaling (p‐p65 and p65), and inflammaroty factor (IL‐1β) in THP‐1 cells (*n* = 3). (d) Immunohistochemistry of Pan‐Kla level in mouse periodontal tissues (*n* = 3). (e) Co‐immunofluorescence of p‐p65 and CD68 in mouse periodontal tissues (*n* = 3). (f) Co‐immunofluorescence of iNOS and CD68 in mouse periodontal tissues (*n* = 3). (g) Three‐dimensional micro‐CT reconstructed images (sagittal view) of maxillae from *Ldha^fl/fl^* and Lysm‐cre; *Ldha^fl/fl^*, followed by quantification of OTM distance (*n* = 5). OTM, orthodontic tooth movement. Data are presented as mean ± SD. ^**^p < 0.01, ^***^p < 0.001, ^****^p < 0.0001.

To validate these findings in vivo, we generated macrophage‐specific *Ldha* conditional knockout mice (LysM‐Cre; *Ldha^fl/fl^*) (Figure S3d). Genotyping and co‐immunofluorescence staining for CD68 and Ldha in periodontal tissues confirmed efficient *Ldha* deletion in macrophages (Figure S3e,f). When subjected to orthodontic force, pan‐lactylation augmentation induced by force was abolished with the lactate level decreased in the periodontal tissue of knockout mice (Figure 3d; Figure S3g). Notably, knockout of macrophage *Ldha* markedly suppressed the proportions of p‐p65+CD68+ and iNOS+CD68+ macrophages in periodontal tissues (Figure 3e,f; Figure S3h). This was associated with diminished NF‐κB signaling and decreased macrophage M1 polarization, indicating attenuated inflammation response. Critically, this correlated with impaired bone remodeling as evidenced by 50% less orthodontic tooth movement (Figure 3g). Collectively, these findings demonstrate that LDHA‐mediated lactylation in macrophages is indispensable for coupling inflammatory progression with adaptive bone remodeling under mechanical compression.

### 2.4 Mechanical Force Potentiates Lactylation of TRAF6

Lactate serves as a substrate for protein lactylation, thereby linking cellular metabolism to immunoregulatory processes. To elucidate the role and underlying mechanisms of lactylation in lactate‐mediated macrophage inflammation under compressive force, we performed integrated proteomic and lactylomic analyses on THP‐1 cells with or without compressive loading using a 4D DIA quantitative proteomics (Figure 4a). In total, 6955 proteins and 18464 lysine lactylation (Kla) sites distributed across 4557 proteins were identified (Figure 4b). Intensity distribution analysis showed comparable average signal intensities for both the proteome and lactylome between control and force‐loaded macrophages, indicating high sample quality (Figure S4a). The frequencies of individual amino acids occurring near lactylation sites were visualized as a heatmap. Lysine (K) residues were enriched in the ‐10, ‐8, and +8 positions, histidine (H) residues in the −5, +1, +2, +3, and +4 positions (Figure 4c). We also used iceLogo to survey amino acids surrounding lactylation sites, and observed similar patterns (Figure S4b). Comparative motif analysis further identified subtle differences in conserved Kla motifs between control and force‐loaded conditions (Figure S4c), indicating force‐dependent remodeling of lactylation patterns. We next analyzed differentially expressed proteins and differentially modified Kla sites. Proteomic analysis identified 378 upregulated and 760 downregulated proteins following force loading (log_2_FC > 0.5, adjusted P < 0.05; Figure 4d). Lactylomic analysis revealed 1604 upregulated and 3272 downregulated Kla sites, corresponding to 1183 and 1782 proteins, respectively (log_2_FC > 0.5, adjusted P < 0.05; Figure 4e). KEGG pathway analyses of differentially expressed proteins showed significant enrichment of metabolic‐related pathways (Figure S4d). Gene Ontology (GO) enrichment analysis further showed enriched ‘oxidative phosphorylation’ and ‘ATP metabolic process’, indicating robust metabolic reprogramming induced by compressive force (Figure S4e). Notably, KEGG pathway analyses of differentially lactylated proteins further supported a central role of lactylation in mediating force‐driven metabolic regulation (Figure S4f). Consistent with this, GO enrichment analysis showed enriched biological processes related to glycolytic reprogramming (Figure S4g). Hierarchical clustering classified lactylated proteins into nine distinct clusters based on changes in protein abundance and lactylation levels (Figure 4f). Among these, Cluster 2 (C2) comprised proteins with unchanged total abundance but markedly increased lactylation, suggesting that lactylation may serve as a key post‐translational mechanism regulating protein function under compressive force. KEGG pathway analysis of C2 proteins revealed significant enrichment in the inflammatory signaling pathway, the toll‐like receptor signaling pathway, which is essential for the activation of the NF‐κB pathway (Figure 4g).

**FIGURE 4:**
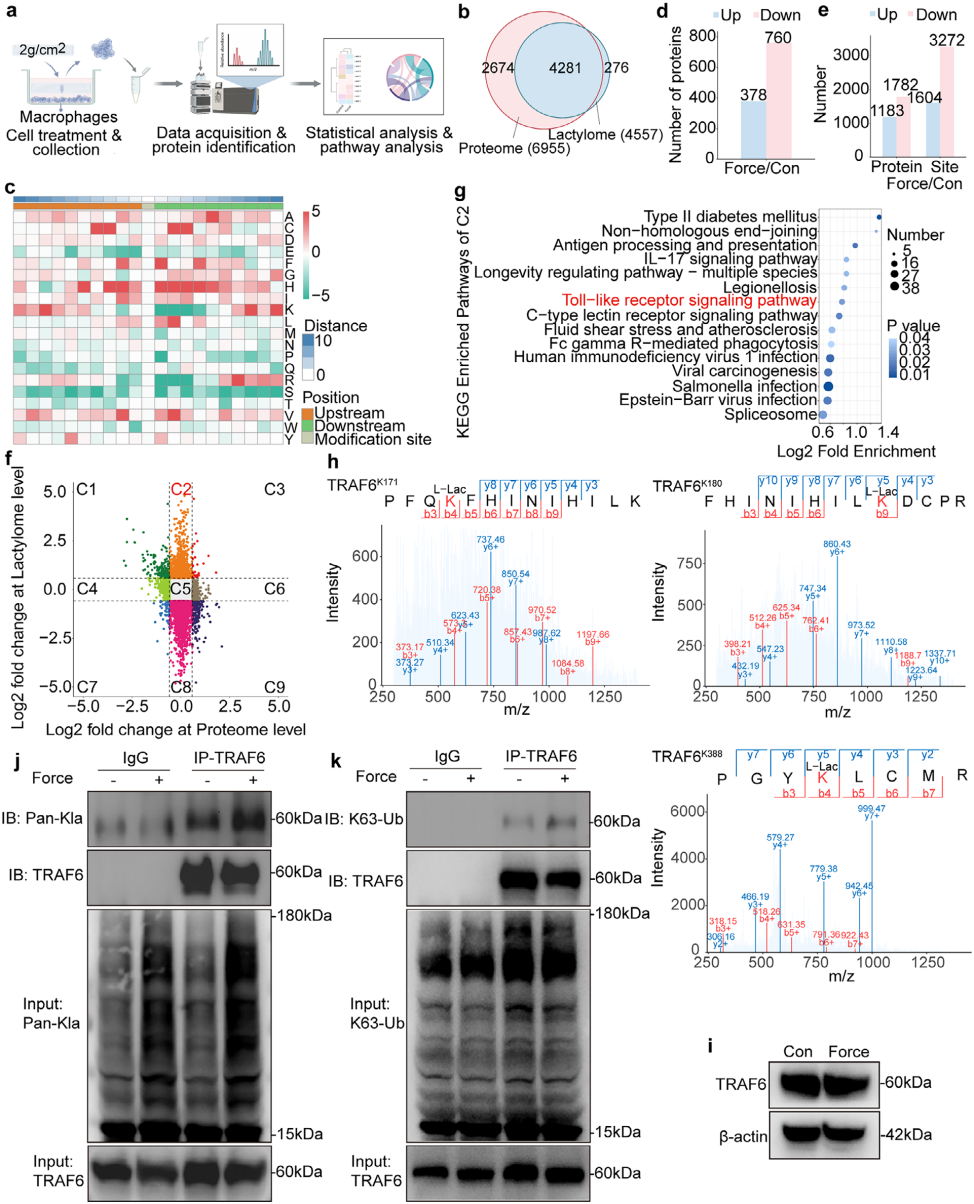
Compressive loading potentiates lactylation of TRAF6. (a) Schematic diagram of the 4D DIA quantitative proteomes and lactylomes of THP‐1 cells with or without compression (2 g/cm^2^). (b) Venn diagram of differentially expressed proteins from quantitative proteomes and lactylated proteins from lactylomes. (c) Heatmap of motif analysis of all identified lactylated proteins. (d) Numbers of differentially expressed proteins from quantitative proteomes (log_2_FC > 0.5, adj *p* < 0.05). (e) Numbers of lactylation (Kla) sites and Kla proteins (log_2_FC > 0.5, adj *p* < 0.05). (f) Hierarchical clustering classified lactylated proteins into nine clusters (C1‐C9) based on changes in protein abundance and lactylation levels (log_2_FC > 0.5, adj *p* < 0.05). (g) KEGG enrichment analysis of proteins in cluster 2 (C2), with unchanged protein abundance but increased lactylation levels. (h) High‐resolution mass spectra identified different lactylated sites of TRAF6 peptides under compressive force. (i) Western blot analysis of TRAF6 in THP‐1 cells with or without compression (*n* = 3). (j, k) THP‐1 cells were subjected to physiological compression or a control condition. Cell extracts were subjected to immunoprecipitation (IP) with the IgG or an antibody against TRAF6. Co‐IP analyses of TRAF6 acetylation (j) and K63‐linked ubiquitination (k) levels were determined.

Given the critical role of NF‐κB pathway activation in macrophage M1 polarization, we focused on key NF‐κB regulators within this cluster (C2). Notably, TRAF6 was identified as a prominently lactylated protein, harboring three upregulated Kla sites (K171, K180, and K388) under compressive loading (Figure 4h). Importantly, we verified that compressive force did not alter total TRAF6 protein levels but significantly increased its overall lactylation (Figure 4i,j). Previous studies have established that NF‐κB activation by TRAF6 depends on its K63‐linked ubiquitination [21]. Consistently, we observed that compressive force markedly enhanced K63‐linked ubiquitination of TRAF6 (Figure 4k). However, whether TRAF6 lactylation directly regulates its K63 ubiquitination remains to be determined.

### 2.5 Mutations of K171 and K180 Lactylation Disrupt TRAF6‐Mediated NF‐κB Signaling Activation and M1 Polarization

In TRAF6, the RING domain together with the first zinc finger (ZF1) domain is essential for its E3 ubiquitin ligase activity [21]. To elucidate how lactylation modulates TRAF6 function, we performed molecular dynamics (MD) simulations to examine the structural dynamics of the RING–ZF1 domains and their interaction with ubiquitin molecules upon lactylation. The crystal structure of the human TRAF6 RING–ZF1 domains (residues 50–211) has been previously reported [22]. Among the lactylation sites identified by mass spectrometry, only K171 and K180 are located within functional domains and are solvent‐accessible on the protein surface. Accordingly, lactylation at K171 and/or K180 was incorporated into the MD models. We compared the structural stability and binding behavior of non‐lactylated TRAF6, mono‐lactylated TRAF6 (K171 or K180), and bi‐lactylated TRAF6 using MD simulations. The results showed that lactylation at K171 conferred enhanced structural stability, as evidenced by the lowest backbone RMSD values (9–12 Å) and the earliest convergence of the TRAF6^K171la^ system (Figure 5a). The radius of gyration (Rg) of both the K171‐lactylated and K171/K180 dual‐lactylated systems remained below 20 Å, markedly smaller than those of the WT and K180‐lactylated systems (Figure 5b), indicating reduced conformational fluctuations and increased compactness. RMSF analysis further revealed that lactylation predominantly affected residues 170–182 within the zinc finger region, which exhibited elevated flexibility (RMSF 4–8 Å) (Figure 5c). Notably, the dual‐lactylated TRAF6^K(171+180)la^ system displayed uniformly lower RMSF values across most residues, reflecting the smallest overall structural fluctuations. Together, these findings indicate that lactylation at K171 stabilizes the RING–ZF1 domains of TRAF6, while dual‐site lactylation may induce a distinct and more stable conformational state.

**FIGURE 5:**
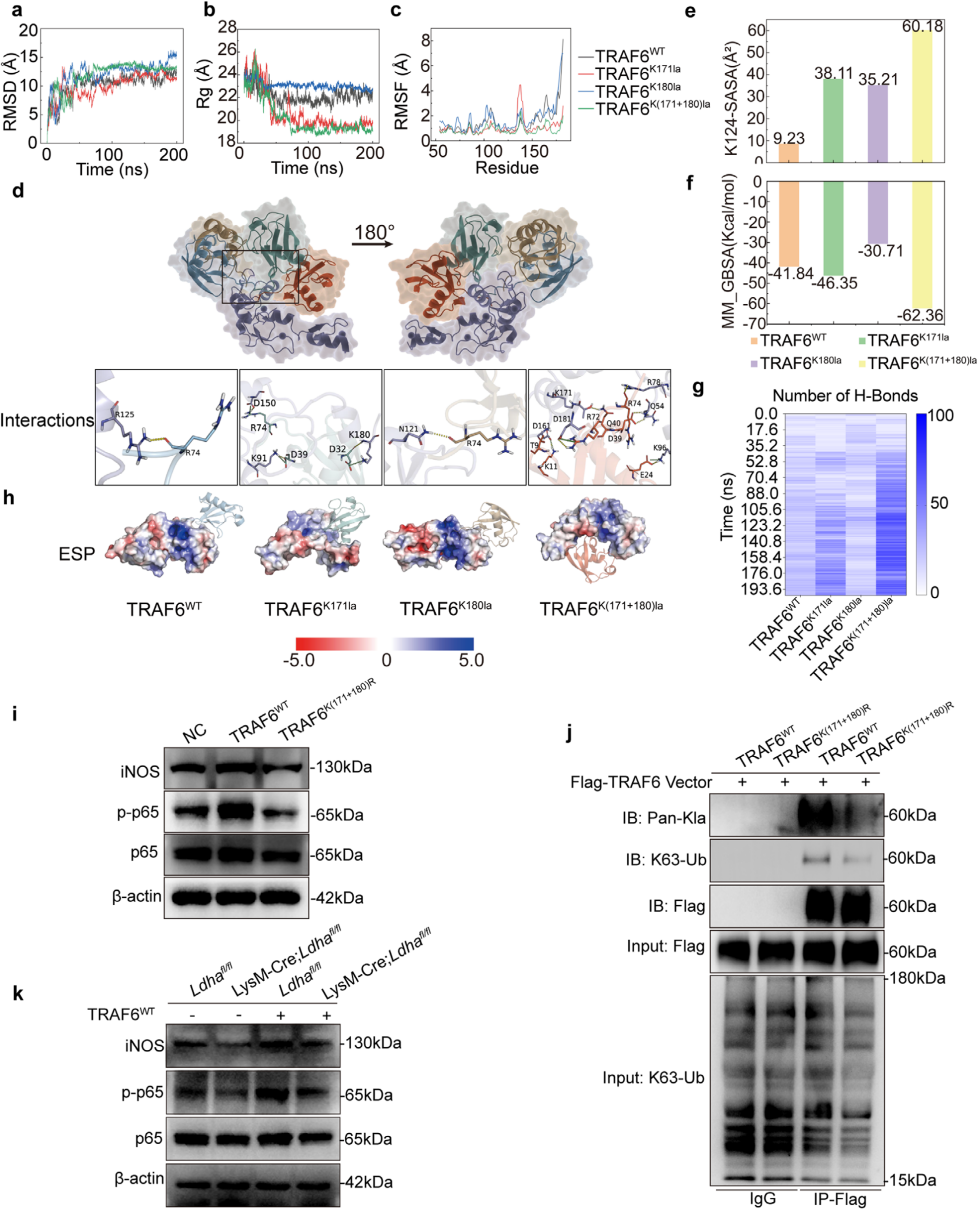
Mutations of K171 and K180 lactylation disrupt TRAF6‐mediated NF‐κB signaling activation and M1 polarization in vitro. (a–c) RMSD (a), Rg (b), and RMSF (c) of TRAF6 wild‐type and its lactylation variants examined by MD simulation. (d) Schematic diagram of representative hydrogen bond (yellow dashed line) or salt bridge (green dashed line) interactions at the TRAF6‐ubiquitin (Ub) binding interface. (e) Bar chart of the solvent accessible surface area (SASA) for the key ubiquitination site K124 of TRAF6. (f) Bar chart of the binding free energy (ΔGbind) for the TRAF6‐Ub complex. (g) Heatmap of the average number of hydrogen bonds at the TRAF6‐Ub interface during the equilibrium phase of simulation. (h) Molecular electrostatic potential surface analysis of TRAF6 wild‐type and its lactylation variants. Red and blue colors represent negative and positive potentials, respectively. (i) Protein levels of iNOS, p‐p65, and p65 in THP‐1 cells transfected with lentivirus (Lv) encoding TRAF6^WT^ or TRAF6^K(171+180)R^ under compressive force. (j) The lactylation and K63‐linked ubiquitination levels of TRAF6^WT^ or TRAF6^K(171+180)R^ in macrophages under compression were determined by co‐immunoprecipitation. (k) Protein levels of iNOS, p‐p65, and p65 in BMDMs with compressive loading.

We next examined the impact of TRAF6 lactylation on ubiquitination at the key site K124, the primary site of K63‐linked ubiquitination on TRAF6 [23]. Analysis of the TRAF6–ubiquitin (Ub) binding interface revealed that the TRAF6^K180la^ system forms only a limited number of hydrogen bond interactions, whereas the TRAF6^K(171+180)la^ exhibits numerous persistent hydrogen bonds and salt bridge interactions (Figure 5d). Solvent‐accessible surface area (SASA) analysis showed that lactylation increased exposure of the ubiquitination site K124, with the dual‐lactylated system displaying the largest value (60.18 Å^2^) (Figure 5e). MM/GBSA calculations demonstrated that lactylation markedly altered the binding affinity between TRAF6 and ubiquitin at the K124 site, with the TRAF6^K(171+180)la^ system exhibiting the lowest binding free energy (−62.36 kcal/mol) (Figure 5f). Statistical analysis of hydrogen bond numbers further confirmed that, at equilibrium, the TRAF6^K(171+180)la^ system exhibits a significantly higher average number of hydrogen bonds than all other systems, whereas the mono‐lactylized K180 system displays the lowest number of hydrogen bonds (Figure 5g). Electrostatic surface analysis further indicated that dual lactylation of K171 and K180 attenuated excessive positive charge at the binding interface, creating a more favorable electrostatic environment for ubiquitin association (Figure 5h).

To validate these findings, we generated lentivirus encoding mutated lactylation of TRAF6 (TRAF6^K(171+180)R^) and conducted functional validations in compressive loading models in vitro (Figure S5a,b). Compared with TRAF6^WT^, TRAF6^K(171+180)R^ markedly decreased the p‐p65/p65 ratio and iNOS expression, indicating suppression of NF‐κB signaling and M1 polarization (Figure 5i; Figure S5c). Consistently, we observed that compressive force markedly enhanced K63‐linked ubiquitination of TRAF6^WT^. However, the enhancement of K63‐linked ubiquitination was suppressed in TRAF6^K(171+180)R^ (Figure 5j). To further determine whether TRAF6 activation depends on lactylation, we conducted rescue experiments using bone marrow‐derived macrophages (BMDMs) from *Ldha^fl/fl^* and LysM‐Cre; *Ldha^fl/fl^* mice. Overexpression of TRAF6^WT^ in *Ldha^fl/fl^* macrophages significantly augmented the force‐induced NF‐κΒ signaling activation and M1 polarization, as evidenced by an increased p‐p65/p65 ratio and elevated iNOS expression. In contrast, this effect was largely abolished in LysM‐Cre; *Ldha^fl/fl^* macrophages (Figure 5k; Figure S5d), indicating that LDHA‐dependent lactate production is required for TRAF6‐mediated NF‐κB activation and M1 polarization under compressive force.

To validate these findings in vivo, mice received periodontal local injection of TRAF6^WT^ or TRAF6^K(171+180)R^, and were subjected to orthodontic force (Figure 6a). The proportions of p‐p65+CD68+ macrophages in periodontal tissue were substantially reduced in TRAF6^K(171+180)R^ group (Figure 6b,c). Critically, this was associated with impaired bone remodeling as evidenced by 50% less orthodontic tooth movement (Figure 6d). Moreover, in *Ldha^fl/fl^* mice, local delivery of TRAF6^WT^ significantly increased the activation of NF‐κB signaling (p‐p65+CD68+) under orthodontic force. However, this effect was abolished in LysM‐Cre; *Ldha^fl/fl^* mice, in which TRAF6^WT^ failed to elevate p‐p65 levels (Figure S5e). In summary, these findings illustrate that lactylation of K171/K180 in TRAF6 in macrophages is essential for activating NF‐κB signaling and M1 polarization under compressive force (Figure 6e).

**FIGURE 6:**
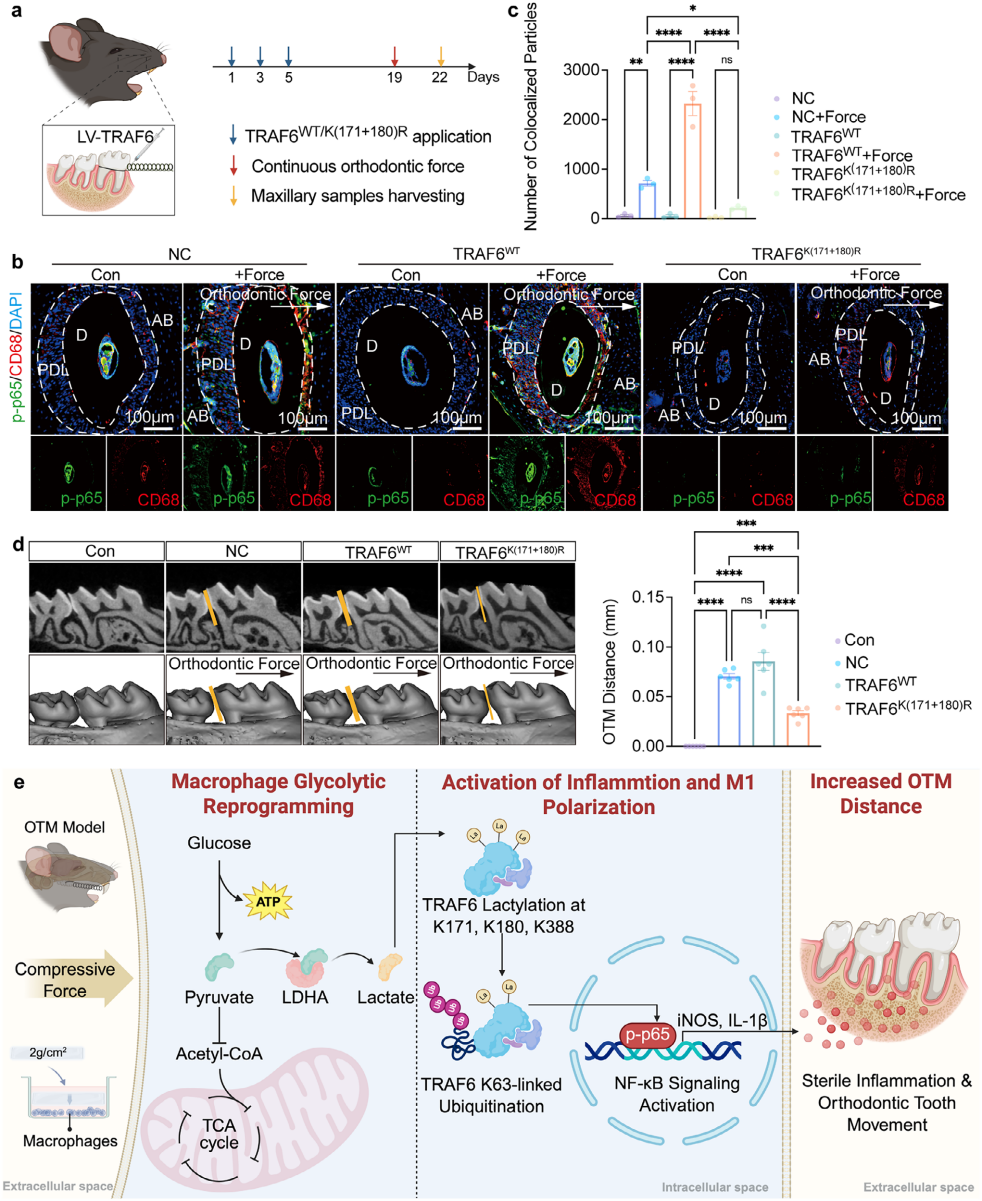
Mutations of K171 and K180 lactylation disrupt TRAF6‐mediated NF‐κB signaling activation and M1 polarization in vivo. (a) Schematic diagram of OTM mouse received periodontal injection of Lv‐TRAF6^WT^/ TRAF6^K(171+180)R^. (b, c) Co‐immunofluorescence revealed the expression of p‐p65 and CD68 in periodontal tissues of controls and mice receiving Lv‐TRAF6^WT^ or Lv‐TRAF6^K(171+180)R^ injection (*n* = 3). (d) Three‐dimensional micro‐CT reconstructed images (sagittal view) of maxillae from different lentivirus‐injected mice, followed by the quantification of OTM distance (*n* = 6). (e) Schematic diagram showing force‐induced lactate‐dependent lactylation of TRAF6 contributes to the activation of NF‐κB signaling and M1 polarization of macrophages, ultimately promoting OTM and bone remodeling. OTM, orthodontic tooth movement. Data are presented as mean ± SD. ^*^p < 0.05, ^**^p < 0.01, ^***^p < 0.001, ^***^ ^*^p < 0.0001.

## 3 Discussion

Metabolic reprogramming is a fundamental mechanism governing immune cell function; however, the role and underlying mechanisms of glycolytic reprogramming in macrophages during OTM have not been elucidated. In this study, we demonstrate that mechanical force induces a shift toward glycolytic metabolism in macrophages, accompanied by increased lactate production. We further show that lactate activates NF‐κB signaling and promotes M1 polarization, thereby enhancing the extent of OTM. Mechanistically, we identify TRAF6 lactylation as a pivotal molecular effector linking mechanical stimulation to macrophage activation during OTM. Lactylation of TRAF6 facilitates K63‐linked ubiquitination, leading to sustained NF‐κB signaling activation. Through molecular dynamics simulations and lentiviral construction of site‐specific TRAF6 lactylation mutants, we demonstrate that dual lactylation at K171 and K180 is essential for regulating K63‐linked ubiquitination, NF‐κB activation, M1 polarization, and subsequent alveolar bone remodeling during OTM. Collectively, our findings establish a mechanistic framework that elucidates macrophage metabolic reprogramming in OTM and reveal how mechanical signals are transduced into intracellular inflammatory signaling, identifying TRAF6 lactylation as a promising therapeutic target for modulating orthodontic tooth movement.

Macrophages are central regulators of innate immunity within the periodontium and play a pivotal role in OTM through their mechanosensitive phenotypic adaptation to compressive force [24]. The functional integration of macrophages within the multicellular periodontal microenvironment is further supported by recent unbiased single‐cell transcriptomic profiling of the OTM immune landscape [25]. Previous studies have demonstrated that macrophage accumulation and activation are essential for force‐induced sterile inflammation and subsequent alveolar bone remodeling during orthodontic treatment [1]. Among the intracellular signaling pathways involved, NF‐κB signaling represents a core regulatory axis controlling macrophage polarization, inflammatory cytokine production, and osteoclast–osteoblast coupling [26]. Activation of NF‐κB signaling in macrophages has been shown to favor M1 polarization, promote pro‐inflammatory cytokine secretion, and accelerate bone resorption—processes that are indispensable for orthodontic tooth movement [27]. Consistent with these reports, we observed robust activation of NF‐κB signaling in macrophages following orthodontic force application, concomitant with enhanced M1 polarization and increased OTM distance. These findings reinforce the concept that NF‐κB signaling serves as a key hub through which macrophages translate mechanical cues into coordinated inflammatory and bone remodeling responses during OTM.

Glycolytic reprogramming, classically known as the Warburg effect in cancer cells, is a metabolic hallmark first described by Otto Warburg in 1924 that maintains homeostasis in proliferating cells by rapidly generating energy and stabilizing redox balance [28]. Increasing evidence supports a role for the Warburg effect beyond cancer, particularly in the development of inflammatory disorders [29, 30]. It is well established that macrophage polarization is tightly coupled to metabolic reprogramming: M1 activation relies on glycolysis, whereas M2 polarization is dependent on oxidative phosphorylation [31]. Chemical signals such as LPS are known to promote macrophage glycolysis via the AKT/mTOR/HIF‑1α pathway, thereby inducing a pro‑inflammatory M1 phenotype [32]. Here, using RNA‐seq, metabolomics, ATP, and lactate determination, we demonstrate that compressive force directly promotes both glycolytic reprogramming, which was accompanied by M1 polarization of macrophages. These findings suggest that metabolic reprogramming constitutes an essential component of mechanical signal transduction in macrophages.

However, the molecular mechanisms linking mechanical force to glycolytic reprogramming remain incompletely understood. Sustained compressive stress during orthodontic tooth movement generates a distinct physical microenvironment characterized by cellular crowding, cytoskeletal deformation, and localized tissue hypoxia. Emerging evidence suggests that cells can sense and transduce mechanical stimuli through multiple structures beyond mechanosensitive ion channels, including the glycocalyx, lipid rafts, caveolae, G protein‐coupled receptors, microvilli, and primary cilia [33, 34]. These mechanosensory systems may converge on signaling pathways that regulate cellular metabolism, ultimately promoting glycolytic activation. Further studies are needed to identify the upstream mechanotransduction pathways responsible for force‐induced metabolic reprogramming in macrophages during OTM.

Glycolytic reprogramming inevitably leads to elevated lactate production. In this study, we observed increased lactate levels in a murine OTM model, in saliva samples from orthodontic patients, and in macrophages subjected to compressive force in vitro. The immunological role of lactate has been controversial, with reports describing both pro‐ and anti‐inflammatory effects depending on the cellular context, disease model, and microenvironment [28]. While some studies suggest that lactate suppresses inflammatory responses and promotes immune tolerance, others have demonstrated that lactate enhances M1 polarization and inflammatory signaling [31, 35, 36]. These discrepancies are likely attributable to differences in experimental systems, lactate concentrations, and cell types examined. In our study, lactate consistently promoted NF‐κB signaling activation and M1 polarization in macrophages, accompanied by increased OTM distance. These findings align with the observed effects of glycolytic reprogramming and support a model in which lactate functions as a pro‐inflammatory signaling metabolite in the mechanical microenvironment of OTM.

LDHA is a critical checkpoint in glycolysis and serves as a pivotal enzyme dedicated to dictating the metabolic flux toward lactate synthesis [37]. Interestingly, although glycolytic activity was markedly increased in macrophages during OTM, LDHA protein expression levels remained mild increase. Previous studies have shown that LDHA can be acetylated, which attenuates its enzymatic function [19, 20]. Because acetyl‐CoA derived from oxidative phosphorylation is a major donor for acetylation, reduced LDHA acetylation may reflect a metabolic shift favoring glycolysis. Consistent with this notion, we observed a significant decrease in LDHA acetylation levels under compressive force, accompanied by a direct increase in its enzymatic activity. The functional requirement for LDHA‐mediated glycolysis in OTM was corroborated by our metabolic blockade experiments. Through myeloid‐specific *Ldha* deletion, we demonstrated that the force‐induced periodontal lactate elevation was substantially suppressed. Consequently, the deletion of *Ldha* in macrophages resulted in attenuated NF‐κB activation, reduced M1 polarization, and a 50% reduction in OTM distance. Collectively, these findings demonstrate that the macrophage LDHA‐lactate axis is indispensable for translating mechanical stimuli into the inflammatory signaling required for bone remodeling. Our results suggest that pharmacological inhibition of LDHA may represent a potential strategy for modulating OTM by suppressing lactate‐driven macrophage activation. Previous studies have shown that the LDHA inhibitor FX‐11 can effectively attenuate lactate‐associated pathological responses in vivo, such as tumor progression and neuroinflammation, supporting the feasibility of targeting LDHA pharmacologically [38, 39]. However, whether localized LDHA inhibition can achieve controllable regulation of orthodontic tooth movement, as well as its long‐term effects on periodontal tissue homeostasis and biosafety, remains to be determined and warrants further investigation.

Protein lactylation, an identified lactate‐derived post‐translational modification, has emerged as a critical molecular bridge linking glycolytic reprogramming to disease progression, particularly in cancer and ischemic disorders [13]. Recent studies have begun to uncover the role of protein lactylation in oral diseases, including lactylation of p300 in periodontal cells and lactylation‐dependent regulation of cementoblast mineralization [37, 40]. These findings highlight the functional relevance of protein lactylation in oral inflammatory and remodeling processes. Additionally, several studies have reported that lactylation could regulate the release of inflammatory factors and polarization of macrophages. For instance, Histone H3 lysine 18 lactylation (H3K18la) might enhance the anti‐inflammatory function of macrophages by inhibiting the expression of inflammatory factors and promoting the expression of Arg1, while the lactylation of PKM2 promotes the transition of pro‐inflammatory macrophages [9, 17]. In the present study, we observed that OTM profoundly altered the global lactylation profile in macrophages, including changes in modification motifs, site abundance, and lactylation intensity. By focusing on proteins whose expression remained stable but whose lactylation levels were markedly altered, KEGG pathway analysis and co‐immunoprecipitation assays revealed a strong enrichment of proteins involved in the TLR–NF‐κB signaling axis.

Building on the identification of TRAF6 as the most intensely lactylated protein in the TLR‐NF‐κB axis under compressive conditions, we further explored the functional implications of this modification. Human TRAF6, also referred to as RNF85, is a member of the TNF receptor‐associated factor (TRAF) protein family that functions as a signal transducer in the NF‐κB pathway, activating IκB kinase (IKK) in response to pro‐inflammatory cytokines and interacting with various protein kinases, including IRAK1/IRAK and SRC [41]. Having demonstrated that compressive force specifically increases TRAF6 lactylation in the macrophages, we then focused on its functional impact. TRAF6 acts as a crucial E3 ligase, and its K63‐linked polyubiquitination is essential for activating the IKK/NF‐κB cascade [42]. Research has indicated that this critical activity depends on its specific domain architecture [21]. TRAF6 features an N‑terminal RING domain followed by four zinc‑finger motifs, which together (residues 50–211) confer its essential E3 ubiquitin ligase activity [22]. Notably, this critical region encompasses the lactylation sites K171/K180 under investigation in the present study. The protein also possesses a centrally located coiled‑coil motif and a C‑terminal TRAF‑C region, both of which are responsible for mediating receptor signaling [43]. Our findings demonstrated that compressive force enhances lactylation at TRAF6 K171/K180. Molecular dynamics simulations revealed that this dual lactylation remodels the protein's conformation and forges a new, stabilized interaction interface with ubiquitin, optimizing electrostatic fit. These results are consistent with a recent work indicating that lactate‐induced SA3K lactylation contributes to its protective effect by promoting protein stability [44]. Guided by this mechanism, we validated that a non‐lactylatable TRAF6 mutant abolishes K63‐linked ubiquitination and subsequent NF‐κB signaling activation. While K‐to‐R mutations can potentially influence protein behavior or function beyond blocking modifications, our MD results and subsequent biochemical assays consistently indicate that the loss of function in the mutant is primarily due to the absence of the lactyl group [44, 45, 46]. Crucially, we observed that the overexpression of TRAF6^WT^ in *Ldha*‐deficient macrophages failed to trigger NF‐κB signaling under pressure, demonstrating that the functional activation of TRAF6 is conditional upon the presence of the LDHA‐mediated lactylation. Critically, in a murine model of OTM, local inhibition of TRAF6 lactylation attenuated both tooth displacement and periodontal inflammation. Specifically, our results confirmed that the inhibition of TRAF6 lactylation reduces the p‐p65/p65 ratio and alleviates inflammation during OTM. Hence, in the present study, compressive force mediates TRAF6 lactylation by regulating LDHA expression and subsequent lactate production.

Several limitations should be acknowledged. First, although we demonstrate a central role for TRAF6 lactylation in macrophage activation during OTM, the mechanosensitive signaling pathways upstream of lactate production and lactylation remain to be elucidated. Second, while our findings highlight macrophages as key mediators, the potential contribution of other immune or stromal cell types to lactate‐dependent signaling during OTM warrants further exploration. Third, we only confirmed the regulatory effect of LDHA via gene knockout mice, and did not conduct localized and controllable animal intervention experiments using LDHA small‐molecule inhibitors. Further pharmacological studies are needed to clarify the bidirectional regulation of tooth movement speed. Finally, long‐term effects and safety considerations of targeting lactylation‐related pathways require careful evaluation before clinical translation.

In summary, our study demonstrates that glycolytic reprogramming functions as a critical signaling conduit through which compressive loading promotes macrophage M1 polarization and ultimately increases the extent of orthodontic tooth movement. Notably, the TRAF6 lactylation axis serves as a central molecular mechanism coupling force‐induced metabolic reprogramming to inflammatory signaling during OTM. Targeting the lactate‐TRAF6 lactylation axis may offer a precise means to control force‐induced sterile inflammation during periodontal tissue remodeling.

## 4 Materials and Methods

### 4.1 Patients and Saliva Samples

To determine salivary lactate levels, unstimulated saliva samples were collected from 20 individuals, including 10 orthodontic patients and 10 age‐ and sex‐matched healthy controls recruited from the Department of Orthodontics, Chongqing, China (September 2023–December 2025). All participants were systemically healthy, caries‐free (DMFT = 0), and exhibited clinically healthy periodontium (shallow probing depth ≤3 mm, absence of bleeding on probing, and minimal clinical attachment loss <1 mm). Controls had no history of orthodontic treatment.

Orthodontic patients received standardized fixed appliance therapy, and a consistent force of 150–200 g was applied for anterior retraction. Saliva was collected after at least 3 months of active treatment (3–6 months). Samples (5 mL per subject) were obtained between 9:00 and 11:00 AM, stored at −80°C, and used for lactate analysis. In compliance with ethical standards for human subjects research, approval for the present investigation was granted by the Ethics Committee of the Affiliated Stomatological Hospital of Chongqing Medical University (Approval No. 2025–080). Additionally, every individual who took part in the study submitted a signed informed consent form prior to their inclusion.

### 4.2 Cell Culture and Compressive Force Application

THP‐1 cells (ATCC TIB‐202) were propagated under standard culture conditions using complete RPMI‐1640 medium containing 10% fetal bovine serum and 1% penicillin‐streptomycin solution, and maintained at 37°C in a controlled atmosphere with 5% carbon dioxide. Cells were stimulated with 100 ng/mL phorbol 12‐myristate 13‐acetate (PMA; HY‐18739, MCE) for 24 hours to trigger differentiation into macrophage‐like cells, leading to an adherent cell ratio of over 95%.

Following differentiation, macrophages were subjected to static compressive loading for 24 h [6] by placing a sterile glass cylinder (diameter: 15 mm; surface area: 1.77 cm^2^) onto the cell monolayer, generating a constant compressive force of 2 g/cm^2^. Lactate intervention was performed using lactic acid (10 mm in vitro and 500 mm in vivo, HY‐B2227, MCE). The pH of the culture medium was balanced and adjusted in each group by sodium hydroxide (NaOH) to exclude pH‐related confounding effects.

### 4.3 Animals and Orthodontic Force Treatment

All animal experiments complied with the updated ARRIVE 2.0 guidelines for preclinical animal studies. The Ethics Committee of the College of Stomatology, Chongqing Medical University (Approval No. 2025–080) authorized all animal procedures. Male C57BL/6 mice aged six weeks (purchased from GemPharmatech Co., Ltd.) and macrophage‐specific *Ldha* knockout mice (LyzM‐Cre; *Ldha^fl/fl^*, Shanghai Model Organisms Center) were housed in a controlled SPF facility with a temperature of 23 ± 2°C, humidity of 40–65%, and a 12/12h light/dark cycle.

Orthodontic force was applied as previously described [47]. Briefly, under avertin anesthesia (DW3101, Shanghai Dowobio Biotechnology Co., Ltd. 20 µL/g), a 0.2 mm ligature wire was used to secure a 0.010‐inch × 6 mm nickel‐titanium closed‐coil spring between the maxillary first molar and incisors, delivering a continuous 20 g mesial force. The appliance was stabilized with light‐cured resin and inspected daily. Maxillary samples were harvested at 1, 3, and 7 days after force application (*n* = 5–6 per group).

### 4.4 Immunofluorescence Double Staining

Periodontal tissue cryosections (8 µm) were processed by fixation, permeabilization, and blocking before immunostaining. Sections were incubated overnight with rabbit‐ and mouse‐derived primary antibodies at 4°C, followed by species‐specific secondary antibodies. Goat anti‐rabbit immunoglobulin G (IgG) conjugated to Alexa Fluor 488, along with goat anti‐mouse IgG conjugated to Alexa Fluor 594, were utilized as secondary antibodies in the experiment. Meanwhile, nuclei were subjected to counterstaining with DAPI, and image acquisition was conducted using a fluorescence microscope. Subsequently, quantitative analysis of double‐positive cells was performed with the aid of ImageJ software. Antibody details are provided in Table S1.

### 4.5 RNA Sequencing

RNA sequencing libraries were constructed and subjected to sequencing by *LC‐Bio Technology Co., Ltd*. (Hangzhou, China), utilizing the Illumina NovaSeq 6000 sequencing platform. TRIzol was used for RNA extraction, and sample quality was examined by Agilent Bioanalyzer 2100, with all samples exhibiting RINs >7.0. Paired‐end 150‐bp sequencing was performed.

Clean reads were mapped to the reference genome of the human, and transcript levels were estimated. Differential expression analysis of genes was conducted with statistical algorithms integrated into DESeq2 and edgeR software. Genes presenting a false discovery rate (FDR) lower than 0.05 and an absolute fold change (|fold change|) of no less than 2 were defined as significantly differentially expressed genes.

### 4.6 Quantitative Metabolomics Analysis

Targeted metabolomic profiling was conducted by *Metabo‐Profile Biotechnology Co., Ltd*. (Shanghai, China) on a UPLC–MS/MS‐based analytical system. Cell samples were rapidly quenched and extracted using pre‐chilled methanol containing internal standards. Following homogenization and centrifugation, supernatants were collected and subjected to chemical derivatization. Derivatized samples were subjected to analysis with an ACQUITY UPLC system connected with a Xevo TQ‐S mass spectrometer (Waters). Metabolites were fractionated on an ACQUITY UPLC BEH C18 chromatographic column with the application of a gradient elution system, while being detected under both positive and negative electrospray ionization (ESI) modes. Quantification was achieved using calibration curves constructed from authentic standards.

Raw data were processed with TMBQ software (Metabo‐Profile) to enable peak integration and metabolite quantification, followed by the performance of multivariate analyses—including principal component analysis (PCA) and orthogonal partial least squares discriminant analysis (OPLS‐DA) —using the iMAP platform (Metabo‐Profile). Metabolites showing high contribution to group discrimination (VIP > 1.0) together with statistical significance were defined as differential.

### 4.7 Intracellular L‐Lactate Dehydrogenase (L‐LDH) Activity Assay

Total intracellular LDH activity was quantified using a commercial L‐LDH Activity Assay Kit (P0393S, Beyotime) according to the manufacturer's protocol. Macrophages were lysed, and the supernatants were incubated with L‐lactate and NAD+ at 37°C. Absorbance was measured at 450 nm. Total protein concentrations were quantified via a BCA kit for normalization. Enzymatic activity was calculated based on the standard curve and expressed as milliunits per milligram of total cellular protein (mU/mg protein).

### 4.8 Lactate Assay

Lactate levels were measured in THP‐1 cell culture supernatants, mouse periodontal tissues, and saliva samples using a commercial lactate assay kit from *Nanjing Jiancheng Bioengineering Institute* (A019‐2‐1). For THP‐1 cells, 5 × 10^6^ cells were seeded per well in six‐well plates, differentiated with PMA, and subjected to static compression for 24 hours before supernatant collection. For mouse samples, approximately 10 mg of periodontal tissue was isolated and homogenized in assay buffer. Saliva samples were thawed at room temperature and centrifuged at 12000 rpm for 10 min at 4°C to remove debris. The concentrations of lactate were assayed following the protocol supplied by the assay manufacturer.

### 4.9 ATP Detection Assay

THP‐1 cells after treatment were subjected to lysis on ice with ATP assay lysis buffer. After centrifugation, supernatants were collected for ATP quantification using an Enhanced ATP Assay kit (Beyotime, S0026). For measurement, 100 µL of ATP working solution was added to a 96‐well plate per well, followed by incubation at 25°C for 3–5 min to deplete background. Each well was added with 20 µL of sample/standard, and luminescence signals (RLU) were immediately measured using a luminometer (EnSpire, PerkinElmer) after mixing.

### 4.10 Acetyl‐CoA Content Assay

Treated THP‐1 cells were lysed in ice‐cold extraction buffer, sonicated, and centrifuged. Supernatants of treated THP‐1 were incubated with a freshly prepared enzyme working solution (Solarbio, BC0980) at 37°C for about 10 minutes. The mixture was analyzed at 340 nm by using a spectrophotometer (Evolution, ThermoFisher). Acetyl‐CoA levels were calculated based on the rate of NADH production (ΔΑ/min), based on the linear correlation between NADH generation rate (ε = 6.22 × 10^3^ L/mol/cm) and Acetyl‐CoA concentration.

### 4.11 Quantitative Real‐Time PCR (qPCR)

Total RNA was extracted with Trizol reagent, reverse‐transcribed to cDNA using a PrimeScript RT kit (Takara, 2680A), and amplified with TB Green Master Mix (Takara, CN830A) containing gene‐specific primers (10 µm each). Reactions (10 µL) underwent 40 cycles of 95°C for 15s and 60°C for 30s on a real‐time PCR system. Gene expression was normalized to GAPDH and calculated via the 2^−ΔΔCt^ method with technical triplicates. Detailed information for all the PCR primers used is provided in Table S2.

### 4.12 Western Blot

Treatment THP‐1 cells were subjected to lysis, and the resulting lysates were centrifuged; the protein concentrations in the obtained supernatants were assayed via the BCA method. Equal quantities of protein (20–40 µg) were subjected to denaturation in Laemmli buffer, fractionated by SDS–PAGE using gradient gels, and then transferred onto PVDF membranes. PVDF membranes were subjected to blocking with 5% skim milk and subsequently incubated with primary antibodies at 4°C overnight, followed by exposure to HRP‐conjugated secondary antibodies at room temperature. Protein signals were detected using enhanced chemiluminescence and quantified with ImageJ. β‐actin served as the internal reference for protein normalization. Antibody details are provided in Table S1.

### 4.13 Co‐Immunoprecipitation (Co‐IP)

Cells were lysed on ice in a non‐denaturing NP‐40/Triton X‐100 buffer containing protease inhibitors. Primary antibodies validated for immunoprecipitation were coupled to Protein A/G magnetic beads at 25°C for 1h, and subsequently incubated with clarified lysates overnight with gentle rotation at 4°C. Washing beads 3–5 times with lysis buffer to remove non‐specific binders, and bound protein complexes were eluted using Laemmli buffer (95°C, 5 min) for subsequent Western blotting or mass spectrometry analysis. Controls included IgG isotype (negative) and input lysate (positive). Antibody details are provided in Table S1.

### 4.14 LC‐MS/MS Analysis

Lysine lactylation proteomic analysis was conducted by *Jingjie PTM BioLab Co., Ltd*. (Hangzhou, China). Proteins were extracted, trypsin‐digested, and lysine lactylation‐modified peptides were enriched using anti‐lactyllysine antibody beads (PTM‐1401RM, PTM Bio).

LC‐MS/MS utilized an EvoSep One system employing the Whisper40SPD separation method, which was coupled online with a timsTOF Pro 2 mass spectrometer operated in dia‐PASEF mode. Subsequent chromatographic separation of peptide samples was achieved via a 21‐minute linear elution gradient, and the eluted peptides were ionized through electrospray ionization (ESI) with an applied voltage set at 1.5 kV.

Data were processed in Spectronaut v18 against the Homo sapiens UniProt database (20231220 release, 20,429 entries) with trypsin/P cleavage (max 4 miscleavages), static carbamidomethylation (Cys), and variable modifications: N‐terminal acetylation, Met oxidation, and L‐lactylation (Lys). PSM/peptide/protein FDR thresholds were set at <1% using reverse decoy databases.

### 4.15 Molecular Dynamics Simulation

The simulation system was constructed based on the TRAF6 N‐terminal domain (residues 54–182, Chain A; PDB ID: 8HZ2) crystal structure. A ubiquitin molecule (PDB ID: 1UBQ) was positioned to model the complex. Using CHARMM‐GUI, we built four systems to compare modification states: non‐lactylated TRAF6 (TRAF6^WT^), mono‐lactylated TRAF6 (TRAF6^K171la^ or TRAF6^K180la^), and bi‐lactylated TRAF6 (TRAF6^K (171+180) la^). Parameters for the lactylated lysine residues and ubiquitin were derived from the additive PTM residue library within the CHARMM36m force field. Simulations were carried out using NAMD 2.14 under the CHARMM36m force field. Systems were immersed in TIP3P water boxes, ion‐neutralized, and equilibrated at physiological salt concentration (pH ∼7.2). Short‐range van der Waals interactions were limited to 12 Å, while long‐distance electrostatics were evaluated using the PME method. Simulations were performed under constant conditions of 300 K and 1 atm regulated by Langevin coupling. Hydrogen‐bond constraints were imposed using the SHAKE method, which permitted a 2 fs timestep. System equilibration followed a standard protocol: 10 000 steps of energy minimization, 250 ps of NVT equilibration with protein backbone restraints, and 250 ps of unrestrained NPT equilibration. Production simulations consisted of three independent 200 ns replicates for each system (total sampling: 2.4 µs). Trajectories were analyzed using VMD and MDAnalysis for RMSD, RMSF, SASA, protein‐protein interaction networks, binding free energy (MM/PBSA), and electrostatic surface potential.

### 4.16 Statistical Analysis

All quantitative data are presented as mean ± standard deviation (SD). GraphPad Prism (version 9.0) was used for statistical analysis. Unpaired two‐sided Student's *t* tests were applied for two‐group comparisons, and one‐ or two‐way ANOVA followed by post hoc analysis was used for multi‐group data. A *P* value <0.05 was considered statistically significant.

## Author Contributions

**H.X**.: contributed to conception, design, and data acquisition, critically revised the manuscript; **Z.G**.: contributed to data acquisition and analysis, critically revised the manuscript; **H.Y., B.W., T.H., Y.S., Y.S., and Z.Y**.: contributed to data acquisition, critically revised the manuscript; **X.X. and Z.L**.: contributed to conception and design, drafted and critically revised the manuscript.

## Conflicts of Interest

The authors declare no conflicts of interest.

## Supporting information




**Supporting File 1**: advs76518‐sup‐0001‐FigureS1‐S5.docx.

**Supporting File 2**: advs76518‐sup‐0002‐TableS1‐S2.docx.

## Data Availability

The data that support the findings of this study are available from the corresponding author upon reasonable request.

## References

[1] Y. Li , Q. Zhan , B. Minyue , Y. Jianru , and Y. Li , “Biomechanical and Biological Responses of Periodontium in Orthodontic Tooth Movement: Update in a New Decade,” International Journal of Oral Science 13, no. 1 (2021): 20, 10.1038/s41368-021-00125-5. PMC8239047 34183652

[2] E. Pastille and A. Konermann , “Exploring the Role of Innate Lymphoid Cells in the Periodontium: Insights into Immunological Dynamics During Orthodontic Tooth Movement,” Frontiers in Immunology 15 (2024): 1428059, 10.3389/fimmu.2024.1428059. PMC11251940 39021572

[3] A. Mukhopadhyay , Y. Tsukasaki , W. C. Chan , et al., “Trans‐Endothelial Neutrophil Migration Activates Bactericidal Function via Piezo1 Mechanosensing,” Immunity 57, no. 1 (2024): 52–67.e10, 10.1016/j.immuni.2023.11.007. PMC10872880 38091995

[4] M. Sauler , J. E. McDonough , T. S. Adams , et al., “Characterization of the COPD Alveolar Niche Using Single‐Cell RNA Sequencing,” Nature Communications 13, no. 1 (2022): 494, 10.1038/s41467-022-28062-9. PMC8789871 35078977

[5] D. He , X. Kou , Q. Luo , et al., “Enhanced M1/M2 Macrophage Ratio Promotes Orthodontic Root Resorption,” Journal of Dental Research 94, no. 1 (2015): 129–139, 10.1177/0022034514553817. 25344334

[6] A. Schröder , P. Käppler , and U. Nazet , “Effects of Compressive and Tensile Strain on Macrophages During Simulated Orthodontic Tooth Movement,” Mediators of Inflammation (2020): 2814015, 10.1155/2020/2814015. PMC7204109 32410848

[7] P. Romani , L. Valcarcel‐Jimenez , C. Frezza , and S. Dupont , “Crosstalk Between Mechanotransduction and Metabolism,” Nature Reviews Molecular Cell Biology 22, no. 1 (2021): 22–38, 10.1038/s41580-020-00306-w. 33188273

[8] H. Tan , S. Wang , X. He , et al., “Microneedles Loaded with Nitric‐Oxide Driven Nanomotors Improve Force‐Induced Efferocytosis Impairment and Sterile Inflammation by Revitalizing Macrophage Energy Metabolism,” ACS Nano 19, no. 9 (2025): 9390–9411, 10.1021/acsnano.5c01877. 40025734

[9] J. Liu , J. Wang , Z. Wang , et al., “PGC‐1α/LDHA Signaling Facilitates Glycolysis Initiation to Regulate Mechanically Induced Bone Remodeling Under Inflammatory Microenvironment,” Bone 185 (2024): 117132, 10.1016/j.bone.2024.117132. 38789096

[10] Z. Zhang , S. Cui , Y. Fu , J. Wang , J. Liu , and F. Wei , “Mechanical Force Induces Mitophagy‐Mediated Anaerobic Oxidation in Periodontal Ligament Stem Cells,” Cellular & Molecular Biology Letters 28, no. 1 (2023): 57, 10.1186/s11658-023-00453-w. PMC10362665 37480044

[11] T. Bertero , W. M. Oldham , E. M. Grasset , et al., “Tumor‐Stroma Mechanics Coordinate Amino Acid Availability to Sustain Tumor Growth and Malignancy,” Cell Metabolism 29, no. 1 (2019): 124–140.e10, 10.1016/j.cmet.2018.09.012. PMC6432652 30293773

[12] B. Li , J. Li , Z. Zhu , et al., “FGF15/FGFR4 Signaling Suppresses M1 Macrophage Polarization and Multi‐Organ Inflammation in Septic Mice by Inhibiting H3K18 Lactylation‐Driven Irf7 Expression through NF2‐Hippo Activation,” Cell Death & Disease 16, no. 1 (2025): 628, 10.1038/s41419-025-07962-w. PMC12361455 40825908

[13] Y. Li , Q. Cao , Y. Hu , et al., “Advances in the Interaction of Glycolytic Reprogramming with Lactylation,” Biomedicine & Pharmacotherapy 177 (2024): 116982, 10.1016/j.biopha.2024.116982. 38906019

[14] M. Schilperoort , D. Ngai , M. Katerelos , D. A. Power , and I. Tabas , “PFKFB2‐Mediated Glycolysis Promotes Lactate‐Driven Continual Efferocytosis by Macrophages,” Nature Metabolism 5, no. 3 (2023): 431–444, 10.1038/s42255-023-00736-8. PMC10050103 36797420

[15] D. Tao , H. Wang , and S. Chang , “Matrix Viscoelasticity Orchestrates Osteogenesis via Mechanotransduction Mediated Metabolic Switch in Macrophages,” in Advanced Healthcare Materials (2025), 2405097, 10.1002/adhm.202405097. PMC12023826 40042258

[16] X. Li , Y. Yang , B. Zhang , et al., “Lactate Metabolism in Human Health and Disease,” Signal Transduction and Targeted Therapy 7, no. 1 (2022): 305, 10.1038/s41392-022-01151-3. PMC9434547 36050306

[17] N. Wang , W. Wang , X. Wang , et al., “Histone Lactylation Boosts Reparative Gene Activation Post–Myocardial Infarction,” Circulation Research 131, no. 11 (2022): 893–908, 10.1161/CIRCRESAHA.122.320488. 36268709

[18] J. Yang , X. Yu , M. Xiao , et al., “Histone Lactylation‐Driven Feedback Loop Modulates Cholesterol‐Linked Immunosuppression in Pancreatic Cancer,” Gut 74 (2025): 1859–1872, 10.1136/gutjnl-2024-334361. PMC12573332 40467104

[19] Q. Ma , Y. Pan , Y. Chen , et al., “Acetylation of Lactate Dehydrogenase Negatively Regulates the Acidogenicity of Streptococcus mutans,” MBio 13, no. 5 (2022): e02013–22, 10.1128/mbio.02013-22. PMC9600946 36043788

[20] D. Zhao , S.‐W. Zou , Y. Liu , et al., “Lysine‐5 Acetylation Negatively Regulates Lactate Dehydrogenase A and Is Decreased in Pancreatic Cancer,” Cancer Cell 23, no. 4 (2013): 464–476, 10.1016/j.ccr.2013.02.005. PMC3885615 23523103

[21] A. J. Middleton , R. Budhidarmo , A. Das , et al., “The Activity of TRAF RING Homo‐ and Heterodimers is Regulated by Zinc Finger 1,” Nature Communications 8, no. 1 (2017): 1788, 10.1038/s41467-017-01665-3. PMC5702613 29176576

[22] Q. Yin , S.‐C. Lin , B. Lamothe , et al., “E2 Interaction and Dimerization in the Crystal Structure of TRAF6,” Nature Structural & Molecular Biology 16, no. 6 (2009): 658–666, 10.1038/nsmb.1605. PMC2834951 19465916

[23] A. K. Singh , S. Umar , S. Riegsecker , M. Chourasia , and S. Ahmed , “Regulation of Transforming Growth Factor β–Activated Kinase Activation by Epigallocatechin‐3‐Gallate in Rheumatoid Arthritis Synovial Fibroblasts: Suppression of K 63 ‐Linked Autoubiquitination of Tumor Necrosis Factor Receptor–Associated Factor 6,” Arthritis & Rheumatology 68, no. 2 (2016): 347–358, 10.1002/art.39447. PMC5383419 26473505

[24] S. Chaushu , Y. Klein , O. Mandelboim , Y. Barenholz , and O. Fleissig , “Immune Changes Induced by Orthodontic Forces: A Critical Review,” Journal of Dental Research 101, no. 1 (2022): 11–20, 10.1177/00220345211016285. 34105404

[25] J. Wang , C. Tao , and H. Liu , “Single‐Cell Atlas of Immune Microenvironment in Orthodontic Tooth Movement,” Journal of Dental Research (2025): 220345251334583, 10.1177/00220345251334583. 40569824

[26] K. M. Sheu and A. Hoffmann , “Functional Hallmarks of Healthy Macrophage Responses: Their Regulatory Basis and Disease Relevance,” Annual Review of Immunology 40 (2022): 295–321, 10.1146/annurev-immunol-101320-031555. PMC10074967 35471841

[27] H. Xu , S. Zhang , and A. A. Sathe , “CCR^2+^ Macrophages Promote Orthodontic Tooth Movement and Alveolar Bone Remodeling,” in Frontiers in Immunology (2022), 835986, 10.3389/fimmu.2022.835986. PMC8854866 35185928

[28] A. Llibre , S. Kucuk , A. Gope , M. Certo , and C. Mauro , “Lactate: A Key Regulator of the Immune Response,” Immunity 58, no. 3 (2025): 535–554, 10.1016/j.immuni.2025.02.008. 40073846

[29] C. M. Krawczyk , T. Holowka , J. Sun , et al., “Toll‐Like Receptor–Induced Changes in Glycolytic Metabolism Regulate Dendritic Cell Activation,” Blood 115, no. 23 (2010): 4742–4749, 10.1182/blood-2009-10-249540. PMC2890190 20351312

[30] A. P. West , I. E. Brodsky , C. Rahner , et al., “TLR Signalling Augments Macrophage Bactericidal Activity through Mitochondrial ROS,” Nature 472, no. 7344 (2011): 476–480, 10.1038/nature09973. PMC3460538 21525932

[31] H. Jiang , H. Wei , H. Wang , et al., “Zeb1‐Induced Metabolic Reprogramming of Glycolysis is Essential for Macrophage Polarization in Breast Cancer,” Cell Death & Disease 13, no. 3 (2022): 206, 10.1038/s41419-022-04632-z. PMC8897397 35246504

[32] S.‐C. Cheng , J. Quintin , R. A. Cramer , et al., “mTOR‐ and HIF‐1α–Mediated Aerobic Glycolysis as Metabolic Basis for Trained Immunity,” Science 345, no. 6024 (2014): 1250684, 10.1126/science.1250684. PMC4226238 25258083

[33] E. K. Mitten and G. Baffy , “Mechanotransduction in the Pathogenesis of Non‐Alcoholic Fatty Liver Disease,” Journal of Hepatology 77, no. 6 (2022): 1642–1656, 10.1016/j.jhep.2022.08.028. 36063966

[34] E. Gordon , L. Schimmel , and M. Frye , “The Importance of Mechanical Forces for In Vitro Endothelial Cell Biology,” Frontiers of Physiology 11 (2020): 684, 10.3389/fphys.2020.00684. PMC7314997 32625119

[35] Y. Fang , Z. Li , L. Yang , et al., “Emerging Roles of Lactate in Acute and Chronic Inflammation,” Cell Communication and Signaling 22, no. 1 (2024): 276, 10.1186/s12964-024-01624-8. PMC11097486 38755659

[36] J. Wang , P. Yang , T. Yu , et al., “Lactylation of PKM2 Suppresses Inflammatory Metabolic Adaptation in Pro‐inflammatory Macrophages,” International Journal of Biological Sciences 18, no. 16 (2022): 6210–6225, 10.7150/ijbs.75434. PMC9682528 36439872

[37] X. Liu , J. Wang , M. Lao , et al., “Study on the Effect of Protein Lysine Lactylation Modification in Macrophages on Inhibiting Periodontitis in Rats,” Journal of Periodontology 95, no. 1 (2024): 50–63, 10.1002/JPER.23-0241. 37436722

[38] Y. Du , Y. Li , F. Ye , et al., “Lactate Exacerbates Neuroinflammation in Sepsis‐Associated Encephalopathy via Promoting Neutrophil Migration from Skull Bone Marrow to the Meninge,” Experimental Neurology 21 (2025): 115318, 10.1016/j.expneurol.2025.115318. 40409661

[39] X. Wei , X. Li , Y. Pan , et al., “LDHA‐Driven Lactate Metabolism Promotes MDSC Activation and Immunosuppressive Microenvironment in Prostate Cancer,” Oncogene (2026): 1544–1556, 10.1038/s41388-026-03737-5. 41922574

[40] Z. Yang , H. Wang , J. Xiao , et al., “KDM6B‐Mediated HADHA Demethylation/Lactylation Regulates Cementogenesis,” Journal of Dental Research 104, no. 1 (2024): 75–85, 10.1177/00220345241286460. PMC11667198 39569625

[41] J.‐J. Lv , H. Wang , C. Zhang , et al., “CD147 Sparks Atherosclerosis by Driving M1 Phenotype and Impairing Efferocytosis,” Circulation Research 134, no. 2 (2024): 165–185, 10.1161/CIRCRESAHA.123.323223. 38166463

[42] Y.‐J. Lim , S.‐A. Park , D. Wang , et al., “MicroRNA‐19b Exacerbates Systemic Sclerosis through Promoting Th9 Cells,” Cell Reports 43, no. 8 (2024): 114565, 10.1016/j.celrep.2024.114565. PMC11440512 39083380

[43] F. Giehler , M. S. Ostertag , T. Sommermann , et al., “Epstein‐Barr Virus‐Driven B cell Lymphoma Mediated by a Direct LMP1‐TRAF6 Complex,” Nature Communications 15, no. 1 (2024): 414, 10.1038/s41467-023-44455-w. PMC10776578 38195569

[44] L. Wang , D. Li , F. Yao , et al., “Serpina3k Lactylation Protects from Cardiac Ischemia Reperfusion Injury,” Nature Communications 16, no. 1 (2025): 1012, 10.1038/s41467-024-55589-w. PMC11760901 39856050

[45] W. Weng , Z. He , Z. Ma , et al., “Tufm Lactylation Regulates Neuronal Apoptosis by Modulating Mitophagy in Traumatic Brain Injury,” Cell Death & Differentiation 32, no. 3 (2025): 530–545, 10.1038/s41418-024-01408-0. PMC11894137 39496783

[46] Z. Yang , C. Yan , J. Ma , et al., “Lactylome Analysis Suggests Lactylation‐Dependent Mechanisms of Metabolic Adaptation in Hepatocellular Carcinoma,” Nature Metabolism 5, no. 1 (2023): 61–79, 10.1038/s42255-022-00710-w. 36593272

[47] S. Wald , A. Leibowitz , Y. Aizenbud , et al., “γδT Cells Are Essential for Orthodontic Tooth Movement,” Journal of Dental Research 100, no. 7 (2021): 731–738, 10.1177/0022034520984774. 33478315

