# Supplementary material for: TRAF6 Lactylation in Glycolytic Macrophages Drives NF‐κB Signaling and M1 Polarization During Orthodontic Tooth Movement
Source: Adv Sci (Weinh). 2026 Jul 14;13(55):e76518. doi: 10.1002/advs.76518 (PMC13366367; doi:10.1002/advs.76518)
Supplement: Supplementary file 1 [file ADVS-13-e76518-s001.docx]

**Supplementary Figures**

**Supplementary Fig. 1** Compressive loading positively regulates inflammation and LDHA activity in macrophages. (**a**) Schematic diagram of RNA sequencing (RNA-seq). THP-1 cells were subjected to control condition or physiological compression (2.0 g/cm²) for 24 h, followed by RNA-seq. (**b**) Heatmap of differentially expressed genes (log₂FC > 0.5, adj P<0.05) from RNA-seq. (**c**) Protein level of iNOS, CD86, p-p65, and p65 of macrophages with or without compressive force (n=3). (**d**) Heatmap of differential metabolites detected by Q300 metabolomic profiling in THP-1 cells were subjected to control condition or physiological compression. (**e**) Quantification of HK2, ALDOC, and LDHA protein levels in macrophages (related to Fig. 1f). (**f**) GSEA analysis of canonical glycolysis and protein acetylation pathways. (**g**) Quantification of Pan-Ace level in THP-1 cells treated with or without compression (related to Fig.1j). **(h**) MS-based analysis of lysine acetylation sites on LDHA protein in THP-1 cells. (**i)** Venn plot of acetylation and lactylation numbers on LDHA protein in from MS of THP-1 cells. (**j**) Three-dimensional micro-CT reconstructed images (sagittal view) of maxillae from different mice with or without orthodontic force application and quantification of the OTM distance (n=6). **(k)** Co-immunofluorescence of p-p65 and CD68 in mice periodontal tissues. **(l)** Co-immunofluorescence of iNOS and CD68 in mice periodontal tissues. OTM, orthodontic tooth movement; MS, mass spectrum. Data are presented as mean ± SD. **p*< 0.05, ***p*< 0.01, ****p*< 0.001, *****p*< 0.0001.

**Supplementary Fig. 2** Lactate positively regulates NF-κB signal pathway and promotes M1 polarization of macrophages. (**a**) Cell viability of macrophages with different lactate concentrationσ application examined by CCK-8. (**b**) Quantification of iNOS, CD86, and p-p65/p65 protein levels in THP-1 cells (related to Fig. 2a). Data are presented as mean ± SD. ***p*< 0.01, ****p*< 0.001.

**Supplementary Fig. 3** *Ldha* knockout in macrophage attenuates NF-κB signaling and M1 polarization during OTM. (**a)** RT-qPCR validation of *LDHA* knockdown efficiency in macrophages by siRNA (n=3). (**b)** The lactate level in THP-1 cells transfected with control siRNA (Con) or si-*LDHA* (n*=*3). (**c)** Quantification of iNOS, CD86, and p-p65/p65 protein levels in THP-1 cells (related to Fig. 3c). (**d, e)** Schematic diagram of the construction of LysM-Cre; *Ldha^fl/fl^* mice (**d**), followed by genotyping (**e**). (**f)** Co-immunofluorescence staining for CD68 and LDHA in periodontal tissues in *Ldha^fl/fl^* and LysM-Cre; *Ldha^fl/fl^* mice. (**g)** The lactate levels in periodontal tissues from *Ldha^fl/fl^* and LysM-Cre; *Ldha^fl/fl^* mice (n*=*6). (**h)** Quantification of p-p65+CD68+ (left) and iNOS+CD68+ (right) macrophages number in mice periodontal tissues (related to Fig. 3e and f). Data are presented as mean ± SD. **p*< 0.05, ***p*< 0.01, *****p*< 0.0001.

**Supplementary Fig. 4** Compressive loading potentiates lactylation of TRAF6. (**a)** Peptide intensity of each group in proteome (left) and lactylome (right) analyses, respectively. (**b)** Icelogo representation showing flanking sequence preferences for Kla sites in all groups. (**c)** Icelogo representation showing flanking sequence preferences of Con group (left) and Force group (right), respectively. (**d, e)** KEGG (**d**) and GO (**e**) enrichment analyses of different expressed proteins from proteome. (**f, g)** KEGG (**f**) and GO (**g**) enrichment analyses of proteins with different lactylation levels.

**Supplementary Fig. 5** Mutations of K171 and K180 lactylation disrupt TRAF6-mediated NF-κB signaling activation and M1 polarization. (**a, b)** Lentivirus encoding TRAF6^WT^ and TRAF6^K(171+180)R^ were successfully constructed (**a**) and successfully transfected in THP-1 cells (**b**). (**c)** Quantification of p-p65/p65 and iNOS protein level in THP-1 cells (related to Fig. 5i). (**d**) Quantification of p-p65/p65 and iNOS protein level in THP-1 cells (related to Fig. 5k). (**e**) Co-immunofluorescence staining for CD68 and p-p65 in periodontal tissues in *Ldha^fl/fl^* and LysM-Cre; *Ldha^fl/fl^* mice with or without the injection of TRAF6^WT^ lentivirus. Data are presented as mean ± SD. **p*< 0.05, ***p*< 0.01, ****p*< 0.001, *****p*< 0.0001.
