# Supplementary material for: TRAF6 Lactylation in Glycolytic Macrophages Drives NF‐κB Signaling and M1 Polarization During Orthodontic Tooth Movement
Source: Adv Sci (Weinh). 2026 Jul 14:e76518. Online ahead of print. doi: 10.1002/advs.76518 (PMC13366367; doi:10.1002/advs.76518)
Supplement: Supplementary file 2 — Supporting File 2: advs76518‐sup‐0002‐TableS1‐S2.docx. [file ADVS-9999-e76518-s001.docx]

**Supplementary Table**

Supplementary Table 1. Antibodies for western blots, immuochemisrtry and immunofluorescence staining.

| Antibodies | Application | Source | Cat. | Company |
| --- | --- | --- | --- | --- |
| β-actin | WB (1:1000) | Rabbit pAb | 20536-1 | Proteintech, China |
| β-actin | WB (1:1000) | Mouse mAb | 66009-1 | Proteintech, China |
| HK2 | WB (1:1000) | Mouse mAb | 66974-1 | Proteintech, China |
| ALDOC | WB (1:1000) | Mouse mAb | 66120-1 | Proteintech, China |
| LDHA | WB (1:1000)  IF (1:100) | Rabbit pAb | 19987-1 | Proteintech, China |
| Flag | WB (1:1000)  IP (1:100) | Mouse mAb | F1804 | Sigma-Aldrich, US |
| Acetylated-Lysine | IP (1:100)  IF (1:100) | Rabbit pAb | 9441 | CST, US |
| Anti-L-lactyllysine | WB (1:1000)  IP (1:100)  IHC(1:100) | Rabbit mAb | PTM-1401RM | PTMbio, China |
| CD68 | IF (1:100) | Mouse mAb | Ab955 | Abcam, UK |
| iNOS | WB (1:1000)  IF (1:100) | Rabbit mAb | 18985-1 | Proteintech, China |
| CD86 | WB (1:1000) | Rabbit mAb | ET1606-50 | HuaBio, China |
| P-NF-κB p65 | WB (1:1000)  IF (1:100) | Rabbit mAb | 3033 | CST, US |
| NF-κB p65 | WB (1:1000) | Rabbit mAb | 8242 | CST, US |
| K63-linkage Specific Ubiquitin Antibody | WB (1:1000) | Rabbit mAb | T56579 | Abmart, China |

Supplementary Table 2. Primers and siRNA sequences.

| Gene | Forward（5‘ to 3’） | Reverse（5‘ to 3’） |
| --- | --- | --- |
| ***Real-time PCR primers*** | | |
| *GAPDH* | GGAGCGAGATCCCTCCAAAAT | GGCTGTTGTCATACTTCTCATGG |
| *IL-1β* | GCCAGTGAAATGATGGCTTATT | AGGAGCACTTCATCTGTTTAGG |
| *iNOS* | CGCATGACCTTGGTGTTTGG | CATAGACCTTGGGCTTGCCA |
| *TNF-α* | AGCCCTGGTATGAGCCCATCTATC | TCCCAAAGTAGACCTGCCCAGAC |
| *IL-6* | CACTGGTCTTTTGGAGTTTGAG | GGACTTTTGTACTCATCTGCAC |
| *IL-10* | GCCGTGGAGCAGGTGAAGAATG | ATAGAGTCGCCACCCTGATGTCTC |
| ***siRNA sequences*** | | |
| *si-LDHA1* | GGAGAAAGCCGUCUUAAUU | AAUUAAGACGGCUUUCUCC |
| *si-LDHA2* | GACUGAUAAAGAUAAGGAA | UUCCUUAUCUUUAUCAGUC |
| *si-LDHA3* | GAUUAAGGGUCUUUACGGA | UCCGUAAAGACCCUUAAUC |
